# HbA1c variability predicts cardiovascular complications in type 2 diabetes regardless of being at glycemic target

**DOI:** 10.1186/s12933-022-01445-4

**Published:** 2022-01-24

**Authors:** Antonio Ceriello, Giuseppe Lucisano, Francesco Prattichizzo, Rosalba La Grotta, Stefan Franzén, Ann-Marie Svensson, Björn Eliasson, Antonio Nicolucci

**Affiliations:** 1 IRCCS MultiMedica, Via Gaudenzio Fantoli, 16/15, 20138 Milan, Italy; 2 CORESEARCH - Center for Outcomes Research and Clinical Epidemiology, Pescara, Italy; 3 Health Metrics, Department of Public Health and Community Medicine, Sahlgrenska Academy, University of Gothenburg, Gothenburg, Sweden; 4 Center for Registries, Västra, Götaland, Sweden; 5 Institute of Medicine, University of Gothenburg, Gothenburg, Sweden

**Keywords:** HbA1c variability, Cardiovascular diseases, Type 2 diabetes, Diabetes complications

## Abstract

**Background:**

HbA1c variability has emerged as risk factor for cardiovascular diseases in diabetes. However, the impact of HbA1c variability on cardiovascular diseases in subjects within the recommended HbA1c target has been relatively unexplored.

**Methods:**

Using data from a large database, we studied 101,533 people with type 2 diabetes without cardiovascular diseases. HbA1c variability was expressed as quartiles of the standard deviation of HbA1c during three years (exposure phase). The primary composite outcome included non-fatal myocardial infarction, non-fatal stroke, all-cause mortality and was assessed during five years following the first three years of exposure to HbA1c variability (longitudinal phase). An expanded composite outcome including non-fatal myocardial infarction, non-fatal stroke, coronary revascularization/reperfusion procedures, peripheral revascularization procedures, and all-cause mortality was also considered, as well as a series of specific cardiovascular complications. Cox models were adjusted for a large range of risk factors and results were expressed as adjusted hazard ratios.

**Results:**

An association between HbA1c variability and all the outcomes considered was found. The correlation between HbA1c variability and cardiovascular complications development was confirmed in both the subgroups of subjects with a mean HbA1c ≤ 53 mmol/mol (recommended HbA1c target) or > 53 mmol/mol during the exposure phase. The risk related to HbA1c variability was higher in people with mean HbA1c ≤ 53 mmol/mol for the primary outcome (p for interaction 0.004), for the expanded secondary outcome (p for interaction 0.001) and for the stroke (p for interaction 0.001), even though HbA1c remained at the target during the follow-up.

**Conclusions:**

These findings suggest that HbA1c variability may provide additional information for an optimized management of diabetes, particularly in people within the target of HbA1c.

**Supplementary Information:**

The online version contains supplementary material available at 10.1186/s12933-022-01445-4.

## Background

High incidence of cardiovascular diseases (CVD) is still present in diabetes [1], and the impact of glycemic control on CVD development is still debated [2]. Growing attention has been recently paid to the possible role of glucose variability (GV) in the development of diabetic complications, particularly cardiovascular ones [3]. GV is defined by the measurement of oscillations in blood glucose or related parameters over a certain interval of time. This description encompasses two main categories of measurements: (1) short-term glucose variability, represented by both within-day and between-day glucose variability, and (2) long-term glucose variability, usually based on serial determinations over a longer period of time using HbA1c [4].

Many observational studies [3] and post-hoc analyses of trials such as the ADVANCE [5], the DEVOTE [6], the VADT [7], the ALLHAT [8], the ACCORD [9], the EMPA-REG OUTCOME [10], the FIELD [11] and the Look AHEAD [12] confirm that in type 2 diabetes (T2D) GV is correlated with an increased risk of CVD and/or all-cause mortality. However, data relative to the impact of long-term GV, assessed as visit-to-visit variability of HbA1c, on a range of cardiovascular outcomes from large, well-characterized, prospective cohorts of patients with T2D, adjusting for multiple risk factors and with proper outcome adjudication, are limited [3]. In addition, recent studies suggest that HbA1c variability, especially HbA1c- coefficient of variation, seems to play an important role in microvascular disease outcomes among patients with relatively optimal baseline glycemic control [13] and in predicting left ventricular remodeling and dysfunction in T2D patients [14]. However, the possible impact of GV on a large range of CV outcomes in patients within the recommended HbA1c target has not been thoroughly explored [3]. The present study evaluated the possible link between visit-to-visit HbA1c variability and the risk of cardiovascular complications among people with T2D and without prevalent CVD at baseline, using data relative to 101,533 patients from the Swedish National Diabetes Register (NDR) [15]. Moreover, to assess the relevance of GV specifically in patients considered at target according to guidelines recommendations [16], we tested the impact of HbA1c variability on the development of CVD comparing patients with a mean HbA1c ≤ 53 mmol/mol with those with a mean HbA1c > 53 mmol/mol.

## Methods

### Population and study design

The database consulted derives from the NDR. The NDR, initiated in 1996, has been described previously [1]. This registry includes information on risk factors, complications of diabetes, and medications for patients 18 years of age or older. All patients have consented to being reported in NDR, while no individual consent is required to be included in this study according to Swedish law. The regional ethical review board approved this study protocol. Around 90% of all patients in Sweden with diabetes are included in NDR [1].

People with T2D and at least five HbA1c measurements, measured by standard procedures, in the NDR between January 1st, 2000, and September 25th, 2019 were considered for this study. Information collected included gender, age, smoking, diabetes duration, measurements of HbA1c, body weight, blood pressure, serum creatinine, urinary albumin excretion, total-cholesterol, low-density lipoprotein cholesterol (LDL), high-density lipoprotein cholesterol (HDL) and triglycerides. Information on antihyperglycemic treatment (diet, oral agents, insulin, oral agents + insulin), antihypertensive treatment (yes vs. no), lipid-lowering treatment (yes vs. no) and aspirin (yes vs. no) was also collected.

The estimated glomerular filtration rare (eGFR) was estimated for each patient by using the Modification of Diet in Renal Disease Eq. (44). Albuminuria from a single measure was categorized as normal (ACR < 30 mg/g), microalbuminuria (30 < ACR < 300 mg/g) and macroalbuminuria (ACR > 300 mg/g). The presence of diabetes complications (retinopathy, cardio-cerebrovascular, heart failure, peripheral arterial disease, minor and major amputations) was also registered, using the International Classification of Diseases, 9th Revision and 10th Revision [17]. The specific codes are listed in Additional file 1: Table S1.

The primary outcome was represented by a composite of first occurrence of non-fatal myocardial infarction, non-fatal stroke, and all-cause mortality. Mortality for specific causes could not be assessed due to the inherent nature of the data contained in the registry.

The following secondary outcomes were considered: non-fatal myocardial infarction, non-fatal stroke, all- cause mortality, coronary reperfusion/revascularization procedures (CABG, PCI), peripheral arterial disease, peripheral vascular angioplasty, hospitalization for heart failure, foot ulcer. An expanded composite outcome (including non-fatal myocardial infarction, non-fatal stroke, coronary revascularization/reperfusion (PCI, CABG) procedures, peripheral revascularization procedures) and all-cause mortality were also considered.

**Fig. 1:**
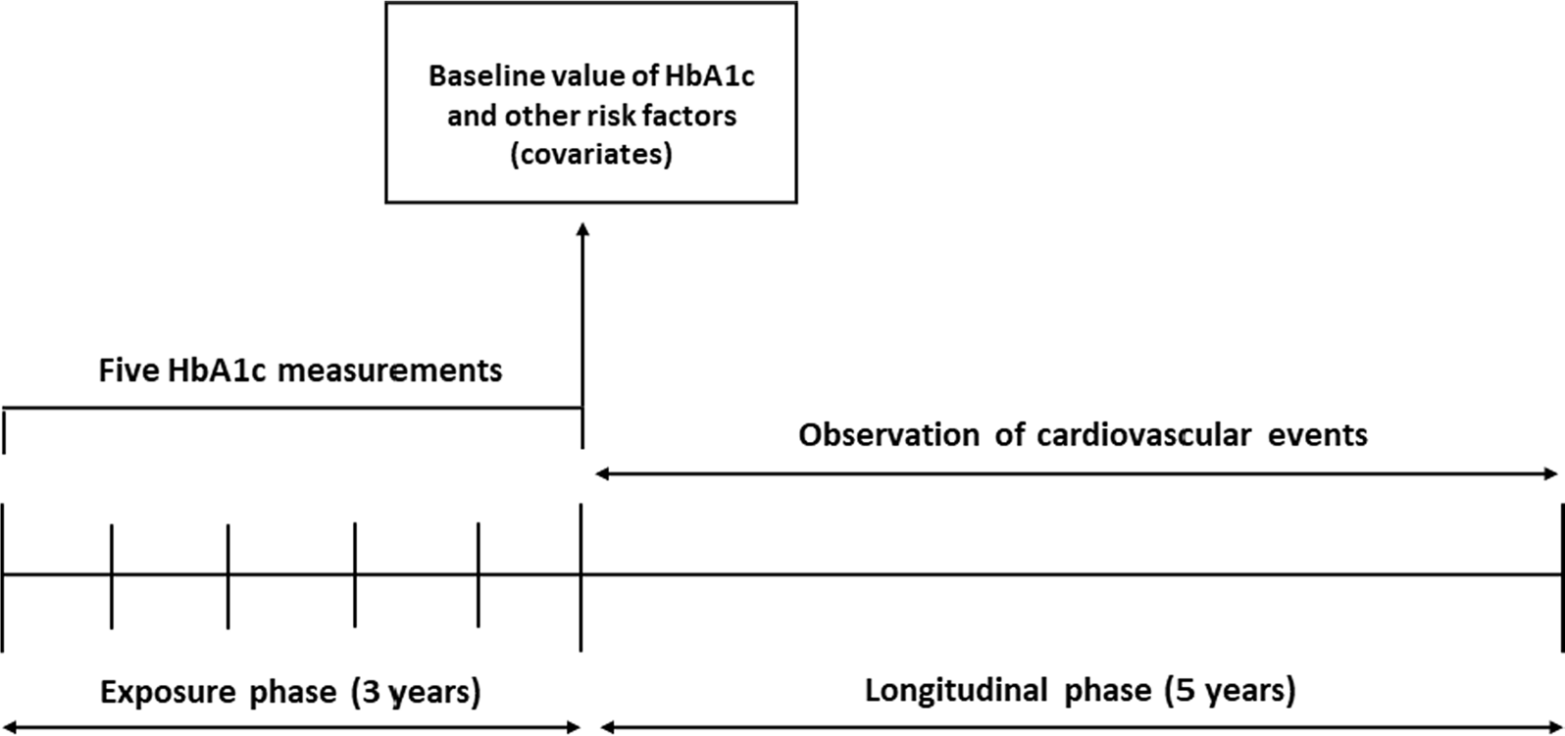
Experimental design

Within the database, we identified all subjects with at least five measurements of HbA1c [18] taken over a period of three consecutive years. Starting from the end of the third year of observation (exposure phase), those with no history of major cardiovascular events were followed up to the latest available data (longitudinal phase) (Fig. 1). Patients with prevalent macrovascular diseases, *i.e*. a previous non-fatal myocardial infarction, non-fatal stroke, coronary reperfusion/revascularization procedures, peripheral arterial disease, peripheral vascular angioplasty, and hospitalization for heart failure, at baseline or experiencing such outcomes during the exposure phase were excluded. Subjects were also divided in two subgroups, based on the average HbA1c levels during the exposure phase ≤ 53 mmol/mol or > 53 mmol/mol.

### Statistical analysis

Descriptive data are summarized as median and interquartile range for continuous variables and percentages for categorical variables. HbA1c variability was expressed as the standard deviation of the measures during the three years preceding the longitudinal phase of the study. A minimum of five measures was considered, in order to have a robust estimate of variability [18]. People were thus grouped according to the quartiles for HbA1c variability. The association between HbA1c variability and risk of developing the outcomes of interest was investigated through multivariate Cox regression analyses. Each Cox model also included the following baseline covariates: age, gender, duration of diabetes, body weight, smoking, values of HbA1c, systolic and diastolic blood pressure, total cholesterol, HDL, LDL, triglycerides, albuminuria, eGFR, retinopathy, treatment for diabetes, hypertension, dyslipidemia, and aspirin. To manage missing data relative to covariates, a category of missing data was added for each covariate in the multivariate analysis. However, such numbers were negligible (data not shown).

In all Cox models, patients were censored at the last visit. Results are expressed as hazard ratios (HRs) with their 95% confidence interval (95%CI).

The association between HbA1c variability and risk of developing the outcomes of interest was also investigated separately for patients with average HbA1c levels ≤ 53 mmol/mol or > 53 mmol/mol during the exposure phase. Quartiles of HbA1c variability were estimated separately for these two subgroups. A test for interaction was applied to assess any differential effect of HbA1c variability in subjects with HbA1c levels ≤ 53 mmol/mol or > 53 mmol/mol during the exposure phase. The predictive impact of being at target of HbA1c or not at the baseline on the outcomes was evaluated by Cox models adjusted for the same risk factors of overall analysis. Tests were 2-sided, and a p value < 0.05 was considered statistically significant. Statistical analyses were performed with SAS software, version 9.4 (SAS Institute Inc. North Carolina, USA).

## Results

From the 515,964 patients with T2D present in the database, we identified all subjects with at least five measurements of HbA1c taken over a period of three consecutive years (n = 310,625 excluded). Starting from the end of the third year of observation (exposure phase), those with no history of major cardiovascular events (n = 83,409 excluded) and with available follow-up (n = 20,397 excluded) were divided into quartiles and followed up to the latest available data (longitudinal phase) (Fig. 1). This design yielded a population of 101,533 subjects without established CVD. Characteristics of patients by quartiles of HbA1c variability are reported in Table 1. People in the upper quartile of HbA1c variability were younger, with a higher prevalence of males, had shorter diabetes duration, a higher prevalence of smokers, and a higher prevalence of insulin use. HbA1c levels increased with increasing levels of HbA1c variability. The median follow-up time of the longitudinal phase was 4·4 years (range 2·1–6·7).

**Table 1:** Characteristics of the study population by quartiles of HbA1c variability

Characteristic	Quartiles of HbA1c variability				p-value
	I	II	III	IV	
No. of patients	25,143	25,442	25,426	25,522	NS
SD of HbA1c	1.9 (1.4–2.3)	3.6 (3.1–4.2)	6.5 (5.6–7.6)	14.3 (11.1–19.7)	< 0.0001
Gender (% males)	47.7	53.5	57.8	63.4	< 0.0001
Age (years)	68.0 (61.0–74.0)	66.0 (58.0–72.0)	63.0 (55.0–71.0)	60.0 (52.0–68.0)	< 0.0001
Smoking %	13.2	14.3	16.0	18.9	< 0.0001
BMI kg/m^2^	28.7 (25.6–32.3)	29.1 (26.1–32.8)	29.6 (26.4–33.3)	29.8 (26.5–33.8)	< 0.0001
Duration of diabetes %					
≤ 2 years	17.2	14.8	13.4	20.5	< 0.0001
2.1–5 years	57.3	54.3	50.0	56.3	
5.1–10 years	14.5	16.0	17.4	12.0	
> 10 years	11.0	15.0	19.2	11.2	
HbA1c (mmol/mol)	45.0 (41.0–49.0)	49.0 (44.0–54.0)	54.0 (47.0–61.0)	54.0 (46.0–67.0)	NS
Systolic blood pressure (mmHg)	135 (125–142)	135 (125–142)	135 (125–142)	133 (125–142)	NS
Diastolic blood pressure (mmHg)	80 (70–82)	80 (70–83)	80 (70–84)	80 (74–85)	NS
Total cholesterol (mmol/l)	4.7 (4.1–5.4)	4.6 (4.0–5.3)	4.6 (4.0–5.3)	4.7 (4.0–5.4)	NS
HDL cholesterol (mmol/l)	1.3 (1.1–1.6)	1.2 (1.0–1.5)	1.2 (1.0–1.4)	1.1 (1.0–1.4)	NS
LDL cholesterol (mmol/l)	2.6 (2.1–3.3)	2.6 (2.0–3.2)	2.6 (2.0–3.2)	2.6 (2.0–3.3)	NS
Triglycerides (mmol/l)	1.4 (1.0–1.9)	1.5 (1.1–2.1)	1.6 (1.2–2.3)	1.7 (1.2–2.4)	NS
Albuminuria %					< 0.0001
No albuminuria	79.8	77.9	74.9	73.9	
Microalbuminuria	11.4	13.4	15.2	16.0	
Macroalbuminuria	2.4	3.3	4.2	4.2	
Not available	6.3	5.5	5.7	6.0	
eGFR (ml/min/1.73m^2^)	79.9 (67.6–93.6)	83.0 (69.6–97.3)	85.6 (71.1–101.0)	89.4 (74.8–105.1)	< 0.0001
Diabetes retinopathy %	12.4%	15.1%	18.8%	18.4%	< 0.0001
Diabetes treatment %					< 0.0001
Lifestyle only	31.8	15.5	8.7	7.5	
Oral agent	60.7	69.6	65.2	61.0	
Insulin	3.2	6.4	9.9	10.8	
Insulin + oral agents	4.3	8.6	16.3	20.6	
Subjects taking GLP-1RA	7	7	9	6	NS
Subjects taking SGLT2-I	1	4	3	8	NS
Antihypertensive medication %	74.7	71.7	69.4	62.6	< 0.0001
Statin medication %	59.5	60.1	58.2	54.4	< 0.0001
Aspirin %	22.2	22.2	22.6	16.4	< 0.0001

### Primary and secondary outcomes in the overall cohort

**Fig. 2:**
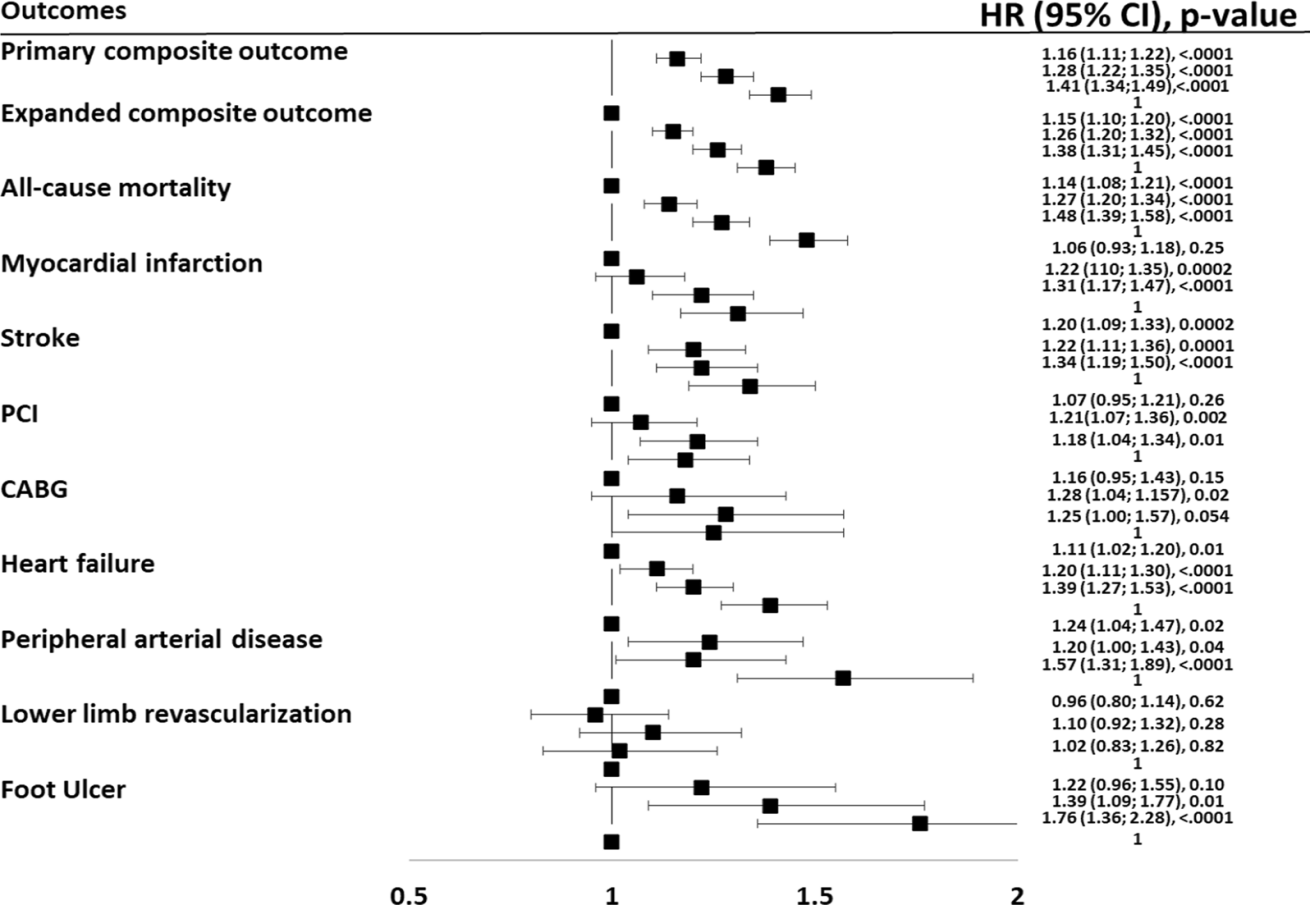
Adjusted hazard ratios (HR) of all the assessed outcomes in the whole population stratified according to quartiles of HbA1c variability. Q1 is the reference group

The association between the measure of intra-individual HbA1c variability and the development of the different outcomes, adjusted for all the available risk factors, is reported in Fig. 2 and in the Additional file 1: Table S2.

The risk of the primary composite outcome represented by non-fatal myocardial infarction, non-fatal stroke, and all-cause mortality significantly increased with increasing HbA1c variability. Compared to the lowest quartile of HbA1c variability, the risk of the primary outcome increased by 16% in the second quartile (HR = 1.16; 95% CI 1.11–1.22), by 28% in the third quartile (HR = 1.28; 95% CI 1.22–1.35), and by 41% in the upper quartile (HR = 1.41; 95% CI 1.34–1.49) (Additional file 1: Table S2, Fig. 2). A linear increase in the risk of event associated with increasing HbA1c variability was documented for most of the outcomes considered (Additional file 1: Table S2, Fig. 2). In particular, compared to people in the lowest quartile of HbA1c variability, those in the upper quartile had a 48% increased risk of all-cause mortality (HR = 1.48; 95% CI 1.39–1.58), a 31% increased risk of myocardial infarction (HR = 1.31; 95%CI 1.17–1.47), a 34% increased risk of stroke (HR = 1.34; 95%CI 1.19–1.50), a 39% higher risk of heart failure (HR = 1.39; 95%CI 1.27–1.53), and a 76% higher risk of foot ulcers (HR = 1.76; 95%CI 1.36–2.28). The risk of the expanded composite outcome increased across quartiles of HbA1c variability, with an excess risk of 38% for the upper quartile compared with the lowest quartile (HR = 1.38; 95%CI 1.31–1.45).

### Subgroup analysis in patients at or not at target

**Fig. 3:**
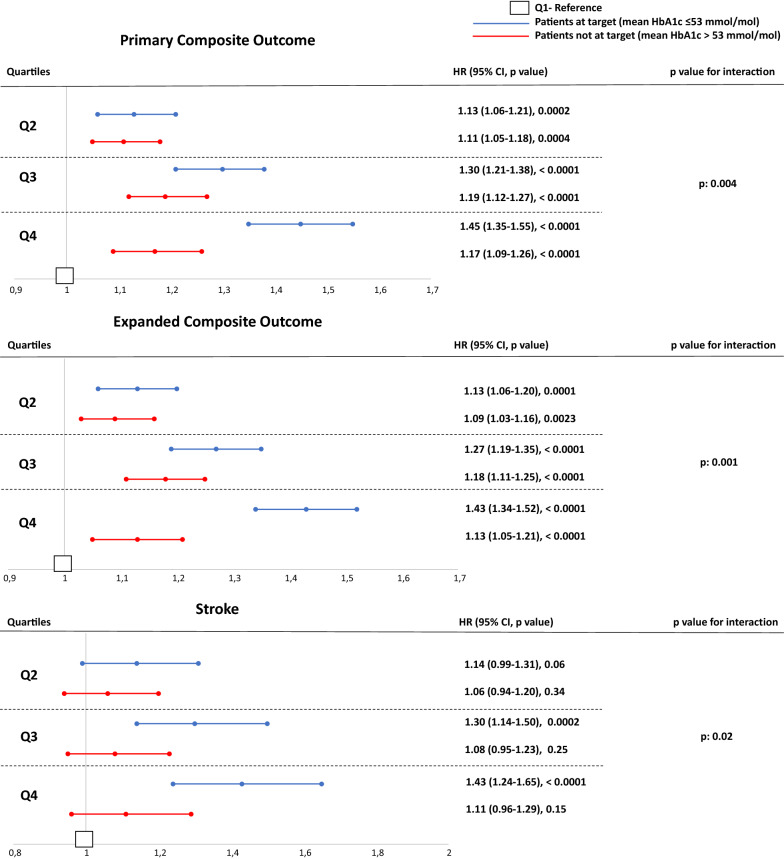
Adjusted hazard ratios (HR) of the significantly different outcomes in the subgroups of patients with a mean HbA1c ≤ 53 mmol/mol (blue lines) or > 53 mmol/mol (red lines) stratified according to quartiles of HbA1c variability. Q1 is the reference group (white squares)

To assess if GV was associated with the development of complications also in subjects at target HbA1c, we analysed and compared outcome data in the subgroups of patients with mean HbA1c ≤ 53 mmol/mol [at target, AT] or > 53 mmol/mol [not at target, NAT] during the exposure phase. Compared to people in the lowest quartile of HbA1c variability, those in the upper quartile and AT had a 45% increased risk for the primary composite outcome, while the upper quartile of NAT group showed a 17% increased risk for the primary composite outcome (HR = 1.45; 95% CI 1.35–1.55 and HR = 1.17; CI 1.09; 1.26, respectively; p for interaction 0.004) (Fig. **3**). The expanded composite outcome showed a similar trend (HR = 1.43; 95% CI 1.34–1.52 for Q4 vs Q1 in AT and HR = 1.13; CI 1.05- 1.21 for Q4 vs Q1 in NAT; p for interaction 0.001) (Fig. 3). Regarding individual outcomes, only stroke showed a significant interaction between AT and NAT subjects (HR = 1.43; 95% CI 1.24–1.65 for Q4 vs Q1 in AT and HR = 1.11; CI 0.96- 1.29 for Q4 vs Q1 in NAT; p for interaction 0.001) (Fig. 3). However, AT subjects in Q4 had a significant higher risk of all-cause mortality, myocardial infarction, hospitalization for heart failure, peripheral artery disease and foot ulcer when compared to Q1 of the same group (Additional file 1: Figure S1). Similar trends but with a generally lower magnitude were observed for NAT patients (Additional file 1: Figure S1).

As shown in Table 2, the mean HbA1c during the follow-up in people AT was below the target in any quartile of HbA1c variability, suggesting that HbA1c levels during the longitudinal phase did not account for the observed differences. In addition, baseline HbA1c was predictive for primary composite outcome (p < 0.001) and for expanded composite outcome (p < 0.001) in NAT subjects but not in people AT (p = 0.13 and p = 0.27, respectively) (data not shown), corroborating the observation that HbA1c variability is linked to the development of complications independently of its levels.

**Table 2:** Mean HbA1c levels and HbA1c variability during the follow-up, according to quartiles (Q1–4) of HbA1c variability at baseline, in subjects with a mean HbA1c ≤ 53 mmol/mol during the exposure phase

	Quartiles of HbA1c variability			
	Q1	Q2	Q3	Q4
N	25,143	25,442	25,426	25,522
HbA1c (mmol/mol)	46.5 (42.0–52.0)	51.3 (45.8–57.6)	56.0 (49.3–63.7)	57.0 (49.3–66.8)
HbA1c SD	2.8 (1.7–4.9)	4.3 (2.6–7.1)	6.0 (3.6–9.1)	6.7 (3.7–10.8)

Data are reported as median and interquartile range. *SD* standard deviation

## Discussion

A number of studies showed an association between HbA1c variability and all-cause mortality [5, 9, 11, 12, 18–21], while the reports on the association between HbA1c variability and cardiovascular complications are quite heterogeneous. Indeed, many studies focused only on some of the possible cardiovascular complications [5, 10–12, 21–24], while others showed no association of HbA1c variability with cardiovascular outcomes [7]. In a meta-analysis, combining data from both type 1 diabetes and T2D, HbA1c variability was superior at predicting diabetes-related complications than mean HbA1c [25]. However, most of these studies had few adjustments for potential confounders, with an inconsistent definition of HbA1c variability [25]. In this study, we showed that high HbA1c variability is predictive of almost all of cardiovascular complication in T2D, demonstrating an association of HbA1c variability with a large range of cardiovascular complications in a very large population of T2D patients in primary CV prevention and longitudinally followed for a long period.

Few studies [18, 26] tried to dissect the contribution of HbA1c variability to the development of complications specifically in patients with HbA1c values within the range recommended by treatment guidelines [16]. Here, we substantiate the evidence that HbA1c variability seems to be more dangerous, at least for the primary outcome, for the expanded composite outcome and for stroke, in people with mean HbA1c at target during the exposure phase, extending previous data derived from much smaller cohorts [18, 26]. Of note, the mean HbA1c level remained at target during the observational follow-up, suggesting that particular attention to HbA1c oscillations should be reserved to people with T2D with good glycemic control. In addition, this observation might provide a further explanation to the observed failure of selected trials testing the effect of a more intensive vs conventional glycemic control on the development of cardiovascular complications [27], such as the ACCORD [28] and the ADVANCE [29] trials, which showed no reduction of cardiovascular events in people treated more aggressively, despite the effective reduction of HbA1c values. Indeed, recent data showed that HbA1c variability combined with mean HbA1c conferred an increased risk for all-cause mortality in the intensive-therapy group in the ACCORD trial [9], while variability in HbA1c alone was independently associated with risk of heart failure in the same trial [30]. Similar findings were observed in the ADVANCE trial [5], albeit no outcome heterogeneity was observed when comparing the intensive and the control group, which however showed similar patterns of HbA1c oscillations. Of note, a high, long-term variability of fasting glucose, but not of HbA1c, was suggested to explain the loss of the beneficial effect of intensive therapy also in the VADT trial [7].

GV is usually defined by the measurement of fluctuations of glucose or other related parameters of glucose homoeostasis over a given interval of time [3]. This description covers two predominant categories of measurements: short-term GV, represented by both within-day and between-day glycemic variability, and long-term GV, based on serial determinations over a longer period of time, usually involving HbA1c, but sometimes serial fasting and postprandial glucose measurements [3]. Short-term GV reasonably cannot be used in long-term studies. The value of a short period of GV clearly cannot be representative of the long period of time, which is needed for the development of the complications. Therefore, visit-to-visit HbA1c variability may represent, at the moment, a good option for evaluating the glucose variability over a given long interval of time [31].

A number of intermediate pathways and mechanisms have been proposed as possible mediators of the GV-induced damage to the cardiovascular system. However, the majority of studies showing an effect of GV on oxidative stress, low-grade inflammation and endothelial dysfunction, three key drivers of all diabetes complications, were conducted with short-term GV [32–36]. Whether such mechanisms are induced also by high visit-to-visit HbA1c variability is unknown [31]. Nevertheless, exposure to intermittent periods of hyperglycemia induces long-lasting epigenetic alterations underlying the chronic activation of oxidative, inflammatory and other detrimental pathways, even when HbA1c is at the target [37, 38]. Alternatively, GV is also associated with a high rate of hypoglycemia, which itself has been suggested as an independent risk factor for both atherosclerosis progression and cardiovascular events [39, 40]. Considering that low levels of HbA1c are frequently accompanied by more frequent episodes of hypoglycemia [41] and that patients admitted to the hospital due to hypoglycemia have higher HbA1c variability [42], the incidence of hypoglycemic episodes could eventually explain the worst effect of HbA1c in people at the target compared to those who are not, a hypothesis deserving exploration in future studies.

Our study has strengths and limitations. Strengths are: the large sample size of people with T2D, the population-based design, minimizing selection bias, the inclusion of people free of cardiovascular complications at the entry and the follow-up duration of median 4.4 years. Limitations are related to the impossibility of establishing whether the correlation between HbA1c variability and CVD is effectively causal. Finally, the very small number of patients treated with GLP-1 or SGLT2-i, drugs able to protect against CVD [43], during the study ruled out that the use of these drugs influenced the results but impede the exploration of whether such drugs benefit also long-term GV.

Specific intervention trials are needed to confirm that reducing GV might reduce the burden of CV complications. However, such trial is very complicated to design and to perform, particularly because to confirm the hypothesis it would be needed that at the end of the study the two compared populations must have the same HbA1c, an equivalent therapy active on cardiovascular risk prevention [43] and differ only for the GV during the study. On the other hand, available evidence suggests that HbA1c should not be the only parameter adopted for a comprehensive evaluation of glycemic control and that careful evaluation of GV might provide additional information for an optimized management of diabetes [44].

## Conclusions

In summary, we provide evidence that HbA1c variability is an independent risk factor for CVD in people with T2D, even when their HbA1c falls within the range recommended by available guidelines.

## Supplementary Information

**Additional file 1.** Additional figures and tables.

## Data Availability

The datasets generated and/or analysed during the current study are not publicly available due to the Swedish legislation, but are available from the corresponding author on reasonable request.

## Abbreviations

95%CI: 95% Confidence interval
CVD: Cardiovascular diseases
eGFR: Estimated glomerular filtration rare
GV: Glucose variability
HRs: Hazard ratios
HDL: High-density lipoprotein cholesterol
LDL: Low-density lipoprotein cholesterol
NDR: Swedish National Diabetes Register
T2D: Type 2 diabetes
HbA1c: Glycated hemoglobin
AT: At-target
NAT: Not-at-target
