# Supplementary material for: HbA1c variability predicts cardiovascular complications in type 2 diabetes regardless of being at glycemic target
Source: Cardiovasc Diabetol. 2022 Jan 24;21:13. doi: 10.1186/s12933-022-01445-4 (PMC8788128; doi:10.1186/s12933-022-01445-4)
Supplement: Supplementary file 1 — Additional file 1. Additional figures and tables. [file 12933_2022_1445_MOESM1_ESM.pdf]

Table S1. **Codes of International Classification of Diseases, 9th Revision and 10th Revision for the outcomes assessed.**

Outcome	ICD9 code (Diagnosis and OP-codes) 1987-1996	ICD10 code (Diagnosis and OP-codes) 1997-2019
**Major cardiovascular events**		
Acute myocardial infarction	*'410'*	*I21…,*
Coronary heart disease	*'410' '411' '412' '413' '414' '*	*I20…, I21…, I22…, I23…, I24…, I25…*
Cardio vascular disease	*410' '431' '432' '433' '434' '436'*	*I21…, I61…, I63…, I64…*
Peripheral arterial disease	*250, 440, 443, 250G, 440C*	*I702, E11.5*
PCI	*3080,*	*FNG0, FNG00, FNG02, FNG05, FNG06, FNG10, FNG30, FNG96*
cabg	*3067, 3127*	*FNA0, FNA00, FNA10, FNA20, FNA96, FNB00, FNB20, FNB96, FNC10, FNC20, FNC30, FNC40, FNC50, FNC60, FNC96, FND10, FND20, FND96, FNE00, FNE10, FNE20, FNE96, FNF00, FNF10, FNF20, FNF30, FNF96*
Stroke	*431' '432' '433' '434' '436'*	*I61…, I63…, I64…*
Heart failure	402, 404, 428	*I50..*
**Diabetes complication**		
Retinopathy	369, 362	H360
Foot ulcer	707, 440, 785, 440, 730, *L98*	*I702C* (Arterial insufficiency ulcer)
amputation	*Not available*	*NHQ09, NHQ11, NGQ09, NGQ11, NGQ19, NGQ99, NFQ09, NFQ19, NFQ99, NEQ19, NEQ99*
Minor amputation	*Not available*	NHQ16, NHQ17
Major amputation	*Not available*	NFQ19, NFQ99, NGQ09, NGQ19, NGQ99, NHQ09, NHQ11, NHQ12, NHQ13, NHQ14, NHQ99
Dialysis	*Not available*	DR015, DR016, DR023, DR024
Peripheral vascular angioplasty_PE	*Not available*	PEA, PEC,PEE,PEF,PEG,PEH,PEL,PEM,PEN,PEP,PEQ,PER,PES,PET,PEU,PEW
Peripheral vascular angioplasty_PE	*Not available*	PFA,PFB,PFC,PFE,PFG,PFH,PFL,PFN,PFP,PFQ,PFR,PFS,PFT,PFU

Table S2 . Average HbA1c levels and HbA1c variability during the follow-up, overall and by average HbA1c level during the exposure phase, according to HbA1c variability quartiles. Data are reported as median and interquartile range.

	Quartiles of HbA1c variability			
	Q1	Q2	Q3	Q4
**Overall population**				
N	25143	25442	25426	25522
HbA1c (mmol/mol)	46.5 (42.0-52.0)	51.3 (45.8-57.6)	56.0 (49.3-63.7)	57.0 (49.3-66.8)
HbA1c SD	2.8 (1.7-4.9)	4.3 (2.6-7.1)	6.0 (3.6-9.1)	6.7 (3.7-10.8)
**HbA1c <= 53 mmol/mol**				
N	14667	14619	14644	14641
HbA1c (mmol/mol)	45.0 (41.0-49.7)	47.4 (43.0-52.3)	49.5 (44.8-54.6)	49.3 (44.0-55.7)
HbA1c SD	2.5 (1.5-4.1)	3.2 (1.9-5.3)	4.2 (2.4-6.7)	4.8 (2.7-4.0)
**HbA1c >53 mmol/mol**				
N	10741	10740	10741	10740
HbA1c (mmol/mol)	59.4 (54.4-65.5)	60.8 (54.8-68.0)	61.3 (54.0-70.0)	57.4 (50.0-67.7)
HbA1c SD	6.0 (4.0-8.8)	6.9 (4.5-10.1)	7.5 (4.7-11.1)	6.7 (3.6-11.3)

Figure S1 . Hazard ratios (HR) for all-cause mortality, myocardial infarction, percutaneous coronary intervention (PCI), coronary artery bypass graft (CABG), peripheral artery disease, hospitalization for heart failure, lower limb revascularization, and foot ulcer, in the subgroups of patients with a mean HbA1c ≤ 7% (grey squares) or > 7% (black squares) stratified according to quartiles of HbA1c variability. Q1 is the reference group.

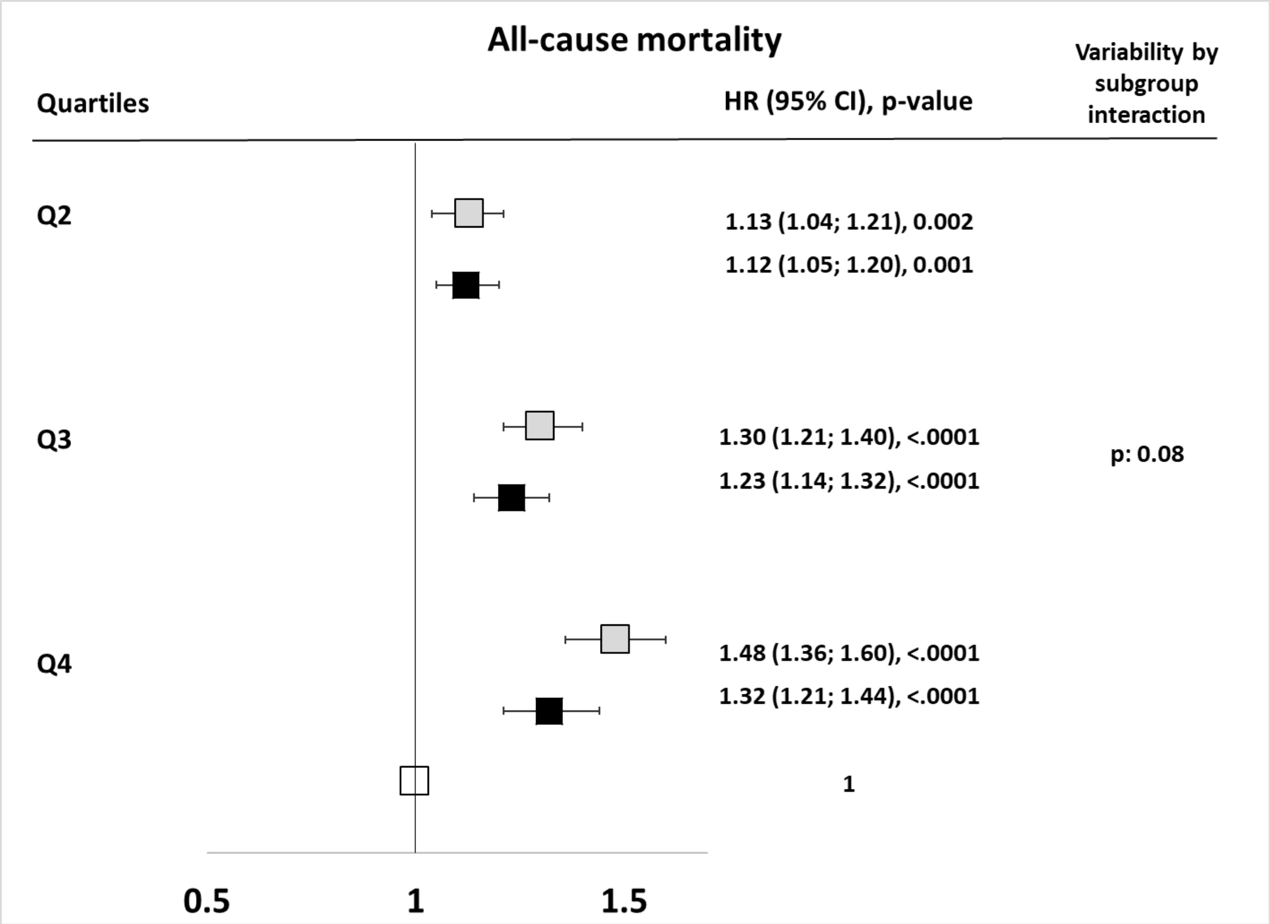

All-cause mortality
Quartiles
HR (95% CI), p-value
Variability by subgroup interaction
Q2
1.13 (1.04; 1.21), 0.002
1.12 (1.05; 1.20), 0.001
Q3
1.30 (1.21; 1.40), <.0001
1.23 (1.14; 1.32), <.0001
p: 0.08
Q4
1.48 (1.36; 1.60), <.0001
1.32 (1.21; 1.44), <.0001
1
0.5
1
1.5

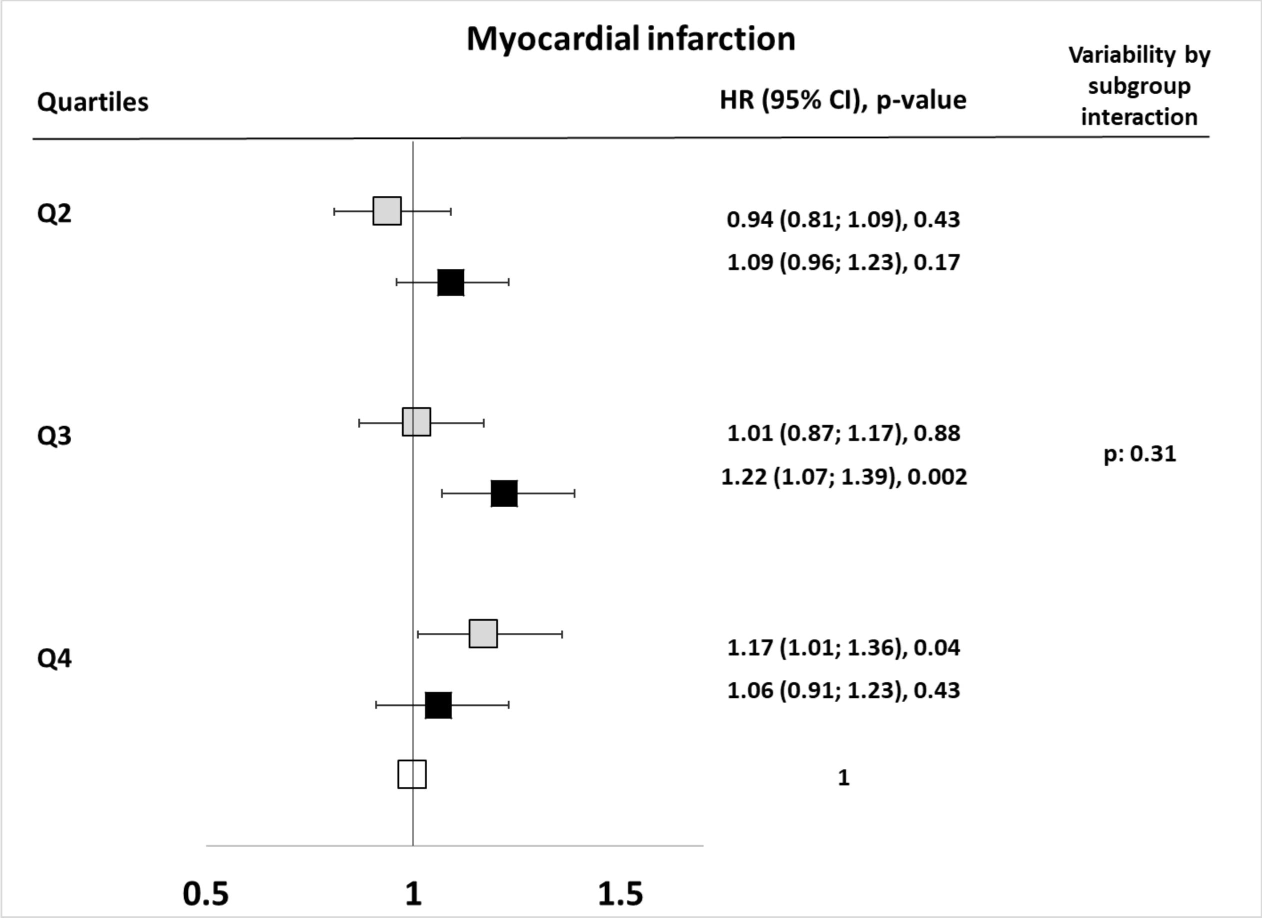

Myocardial infarction
Quartiles
HR (95% CI), p-value
Variability by subgroup interaction
Q2
0.94 (0.81; 1.09), 0.43
1.09 (0.96; 1.23), 0.17
Q3
1.01 (0.87; 1.17), 0.88
1.22 (1.07; 1.39), 0.002
p: 0.31
Q4
1.17 (1.01; 1.36), 0.04
1.06 (0.91; 1.23), 0.43
1
0.5
1
1.5

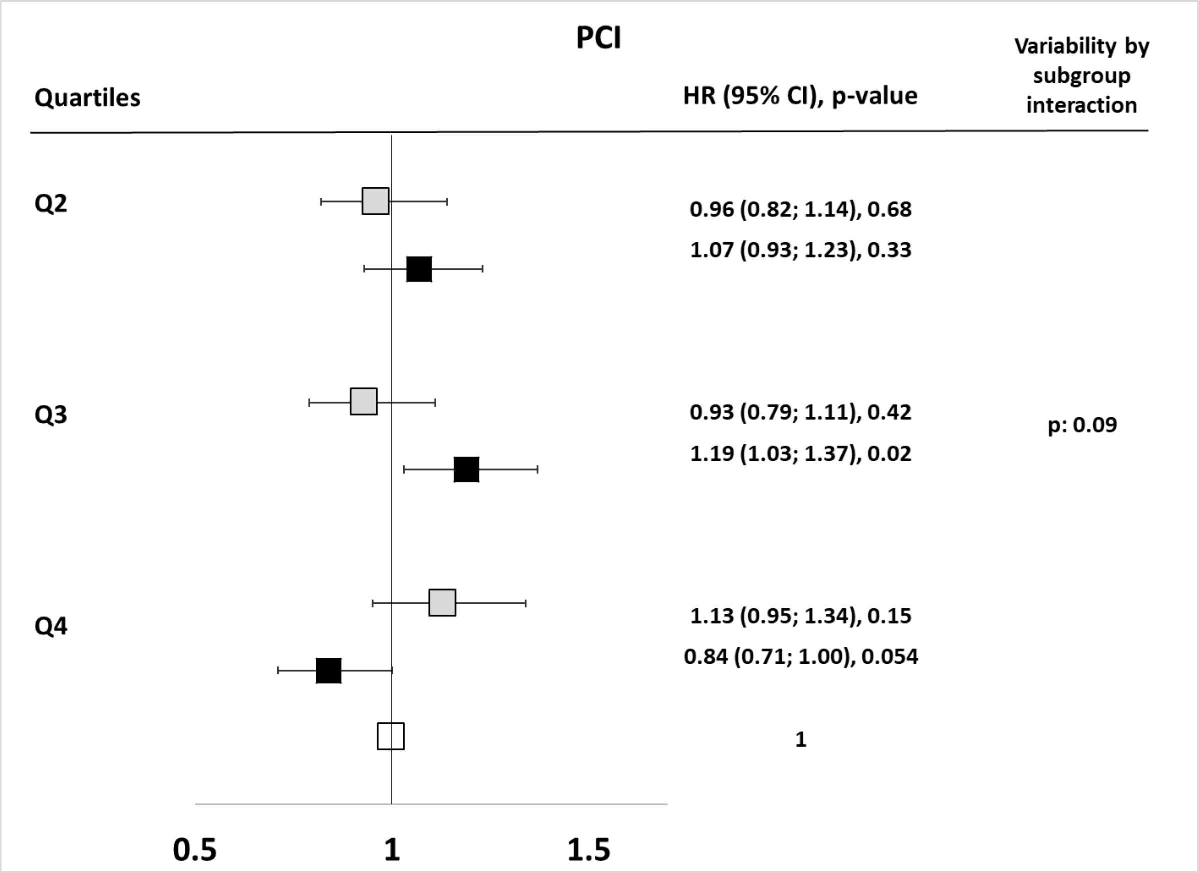
PCI
Quartiles
HR (95% CI), p-value
Variability by subgroup interaction
Q2
0.96 (0.82; 1.14), 0.68
1.07 (0.93; 1.23), 0.33
Q3
0.93 (0.79; 1.11), 0.42
1.19 (1.03; 1.37), 0.02
p: 0.09
Q4
1.13 (0.95; 1.34), 0.15
0.84 (0.71; 1.00), 0.054
1
0.5
1
1.5

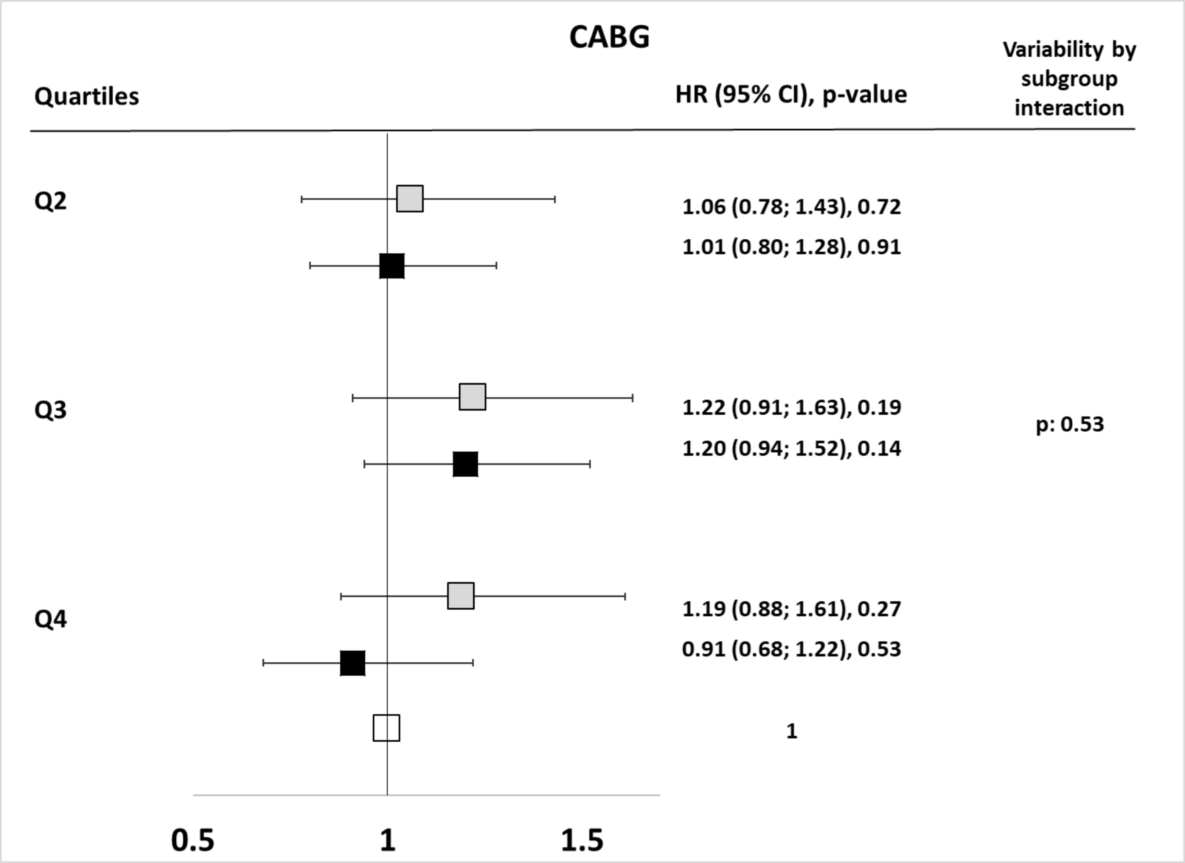
CABG
Quartiles
HR (95% CI), p-value
Variability by subgroup interaction
Q2
1.06 (0.78; 1.43), 0.72
1.01 (0.80; 1.28), 0.91
Q3
1.22 (0.91; 1.63), 0.19
1.20 (0.94; 1.52), 0.14
p: 0.53
Q4
1.19 (0.88; 1.61), 0.27
0.91 (0.68; 1.22), 0.53
1
0.5
1
1.5

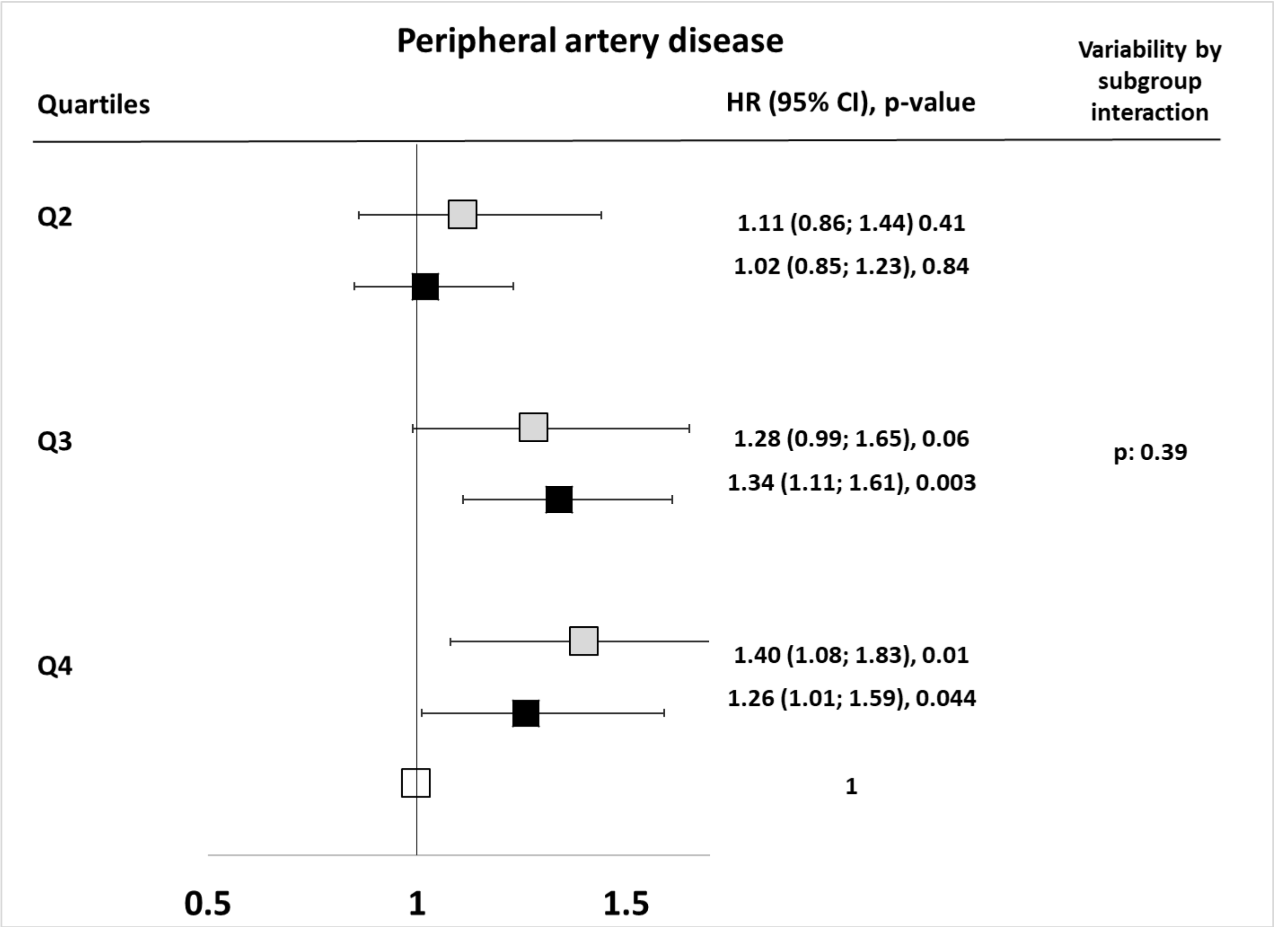
Peripheral artery disease
Quartiles
HR (95% CI), p-value
Variability by subgroup interaction
Q2
1.11 (0.86; 1.44) 0.41
1.02 (0.85; 1.23), 0.84
Q3
1.28 (0.99; 1.65), 0.06
1.34 (1.11; 1.61), 0.003
p: 0.39
Q4
1.40 (1.08; 1.83), 0.01
1.26 (1.01; 1.59), 0.044
1
0.5
1
1.5

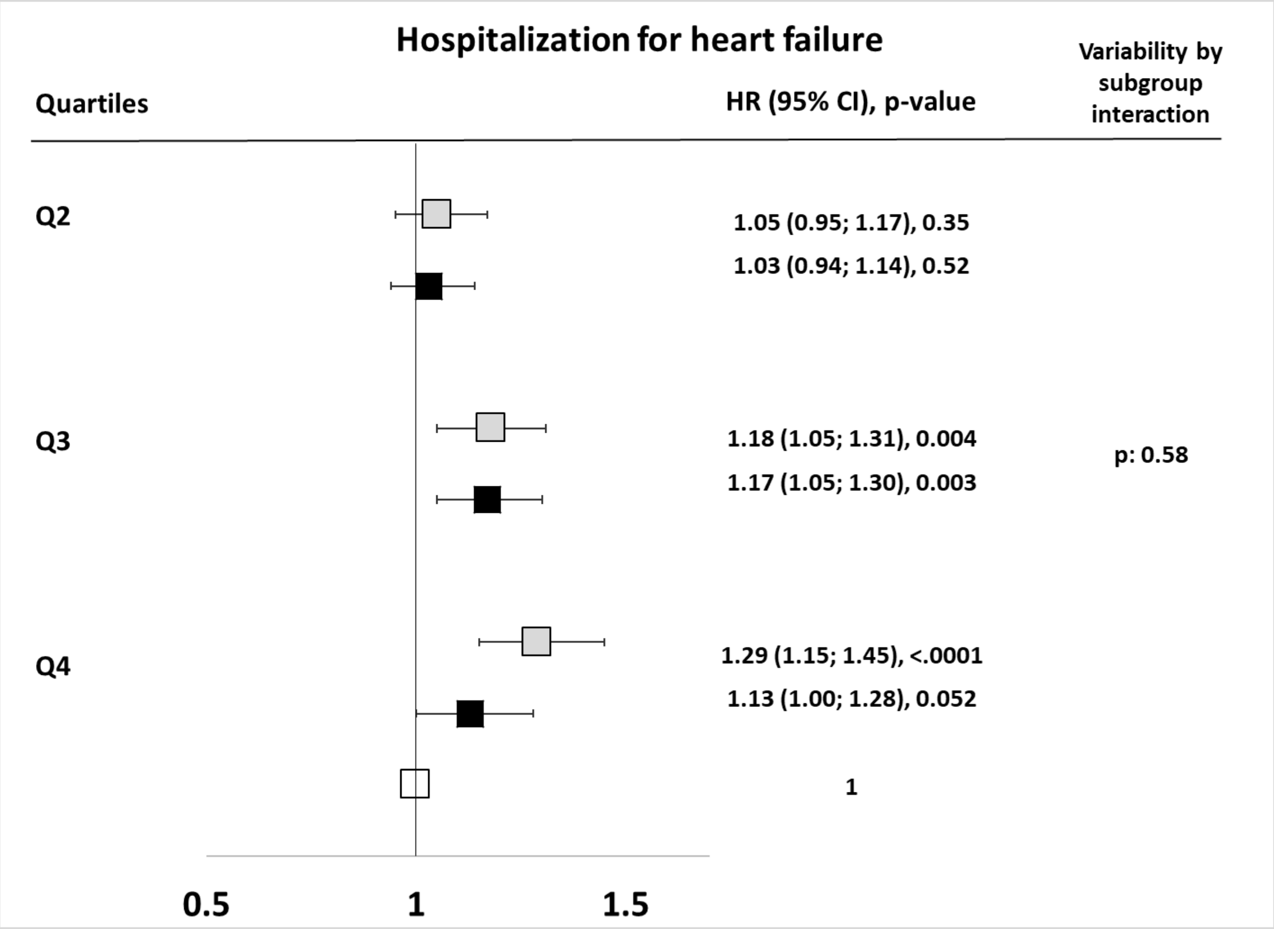
Hospitalization for heart failure
Quartiles
HR (95% CI), p-value
Variability by subgroup interaction
Q2
1.05 (0.95; 1.17), 0.35
1.03 (0.94; 1.14), 0.52
Q3
1.18 (1.05; 1.31), 0.004
1.17 (1.05; 1.30), 0.003
p: 0.58
Q4
1.29 (1.15; 1.45), <.0001
1.13 (1.00; 1.28), 0.052
1
0.5
1
1.5

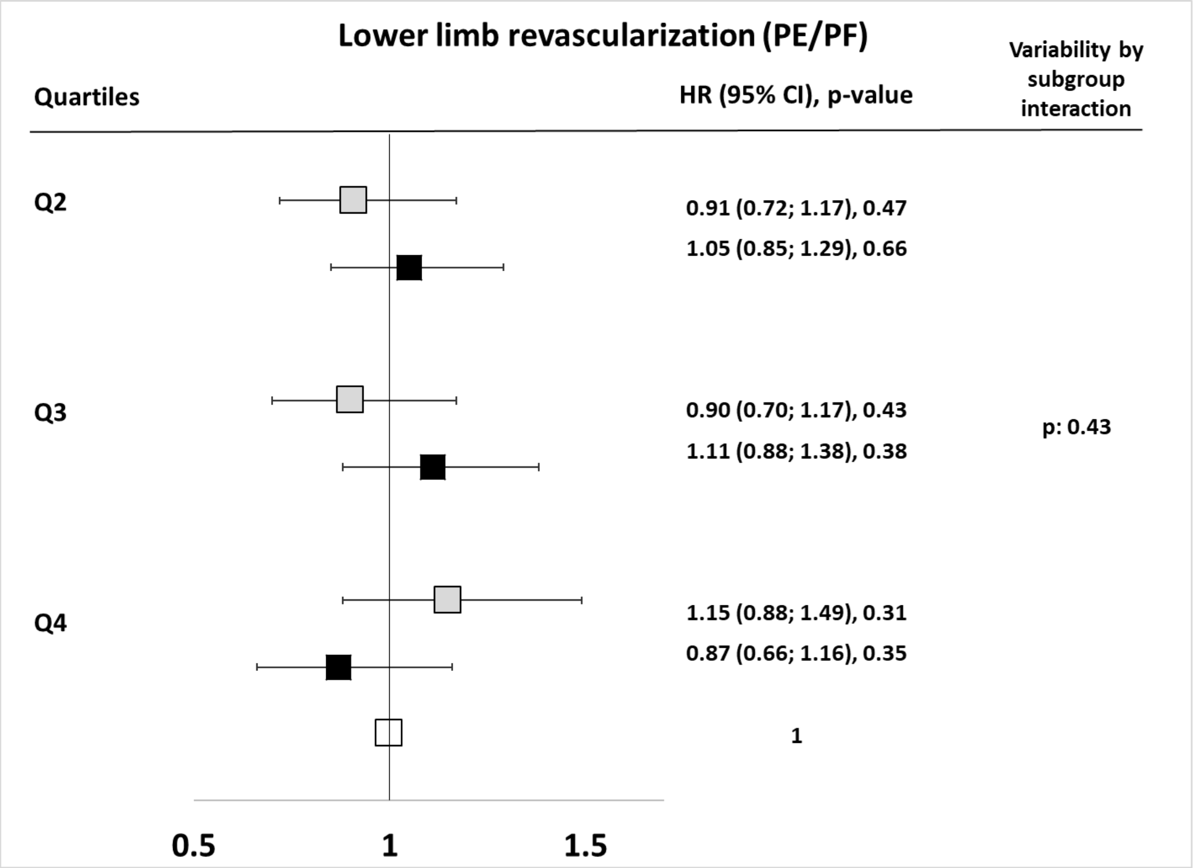
Lower limb revascularization (PE/PF)
Quartiles
HR (95% CI), p-value
Variability by subgroup interaction
Q2
0.91 (0.72; 1.17), 0.47
1.05 (0.85; 1.29), 0.66
Q3
0.90 (0.70; 1.17), 0.43
1.11 (0.88; 1.38), 0.38
p: 0.43
Q4
1.15 (0.88; 1.49), 0.31
0.87 (0.66; 1.16), 0.35
1
0.5
1
1.5

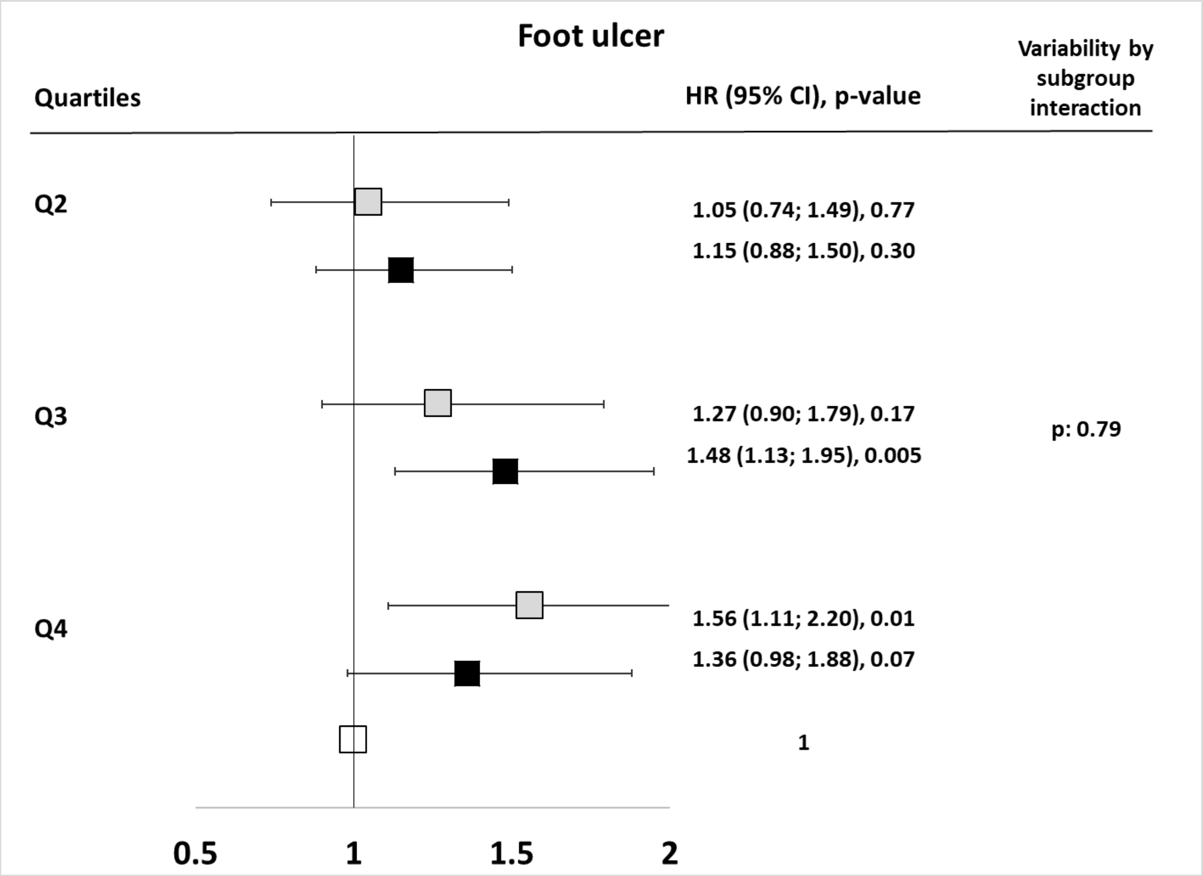
Foot ulcer
Quartiles
HR (95% CI), p-value
Variability by subgroup interaction
Q2
1.05 (0.74; 1.49), 0.77
1.15 (0.88; 1.50), 0.30
Q3
1.27 (0.90; 1.79), 0.17
1.48 (1.13; 1.95), 0.005
p: 0.79
Q4
1.56 (1.11; 2.20), 0.01
1.36 (0.98; 1.88), 0.07
1
0.5
1
1.5
2
